# Empagliflozin and Arterial Stiffness in Patients with Type 2 Diabetes: A Real-World Case-Control Study

**DOI:** 10.2174/0118715303372020250131060159

**Published:** 2025-02-18

**Authors:** Francesco Tassone, Cinzia Ferreri, Arianna Rossi, Giorgio Borretta, Guido Pastorini, Fabio Anastasio, Mauro Feola

**Affiliations:** 1 Diabetes Division, Ospedale Maggiore di Chieri, Chieri, Italy;; 2 Internal Medicine Ospedale di Saluzzo, Saluzzo, Italy;; 3 Internal Medicine Ospedale Moncalieri, Moncalieri, Italy;; 4 Division of Endocrinology, Diabetes & Metabolism, Santa Croce e Carle Hospital, Cuneo, Italy;; 5 Cardiology Division, Ospedale Regina Montis Regalis, Mondovi, Italy

**Keywords:** Empagliflozin, arterial stiffness, type 2 diabetes mellitus, pulse wave velocity, ventricular function, albumin to creatinine ratio, endothelial function

## Abstract

**Background:**

Sodium-glucose cotransporter-2 (SGLT2) inhibitors have demonstrated beneficial cardiovascular and renal effects in patients with type 2 diabetes mellitus (T2DM).

**Objective:**

The objective of this case-control study was to evaluate the efficacy of empagliflozin in modifying the arterial stiffness in type 2 diabetic patients.

**Methods:**

Pulse wave velocity (PWV) and other parameters of arterial stiffness were assessed at baseline and after three months of empagliflozin treatment in 16 consecutive outpatients with type 2 diabetes mellitus (T2DM) exhibiting normal left ventricular function and no signs of heart failure. A control group of 16 T2DM outpatients not treated with SGLT2 inhibitors was used for comparison.

**Results:**

Duration of diabetes mellitus and sex distribution did not differ between groups. Patients in the empagliflozin group were younger compared to controls (64.1 ± 8.68 *vs* 74.45 ± 8.13, *p* < 0.05). At 3-month follow-up, empagliflozin treatment significantly reduced HbA1c (7.9 ± 0.78 *vs* 7.04 ± 1.09%, *p* < 0.008). Empagliflozin significantly improved PWV compared to controls (from 13.2 ± 2.0 m/sec to 12.3 ± 1.8 m/sec; *P* = 0.001; in the control group 12.8 ± 2.3m/s to 13.2 ± 2.4, *p* = ns, with age and HbA1c as covariates) as well as body weight that significantly reduced (86.75 ± 16.16 kg *vs* 81.71 ± 16.5 kg, *p* =0.001) and BMI (30.48 ± 5.4 versus 28.75 ± 5.66 kg/m2, *p* < 0.002) in comparison to controls. Estimated glomerular filtration rate (eGFR) remained unchanged whereas a significant improvement of urine Albumin to Creatinine ratio with empagliflozin emerged (17.8 ± 46.8 *vs* 12.2 ± 35.7 mg/mmol, *p* = 0.049).

**Conclusion:**

In this clinical study, mid-term treatment with empagliflozin in patients with type 2 diabetes mellitus (T2DM) resulted in a significant reduction in arterial stiffness. Additionally, the improvement in the urine albumin-to-creatinine ratio suggests a potential enhancement in endothelial function.

## 1 INTRODUCTION

Type 2 Diabetes Mellitus (T2DM) is a chronic metabolic disorder that poses a significant risk factor for cardiovascular disease (CVD), which appears to be the leading cause of death among individuals with T2DM [1]. Sodium-glucose cotransporter 2 inhibitors (SGLT2 inhibitors) represent a new class of oral agents for lowering blood glucose levels that block SGLT2 cotransporters on the luminal surface of the proximal renal tubule, increasing glycosuria and natriuresis. This mechanism of action leads to a reduction in glycosylated hemoglobin, body weight, blood pressure, and albuminuria [1-6].

Large randomized controlled trials and associated meta-analyses have shown that SGLT2 inhibitors provide significant benefits in reducing atherosclerotic major adverse cardiovascular events, particularly in patients with established atherosclerotic cardiovascular disease. Additionally, these studies revealed a reduction in hospitalizations for heart failure and a slowdown in the progression of renal disease, regardless of the presence of existing atherosclerotic cardiovascular disease or a history of heart failure [5, 6]. The exact mechanisms behind these cardioprotective effects are not yet fully understood. However, one commonly referenced factor is the proposed impact on arterial stiffness and endothelial dysfunction [3, 4].

Arterial stiffness, measured by pulse wave velocity (PWV), has been demonstrated to be a robust independent predictor of cardiovascular events [7]. Recently, it has been acknowledged as the gold standard non-invasive method for assessing large artery stiffness, as outlined in the Consensus Document on Ventricular-Arterial Coupling in Cardiac Disease [8].

The effect of SGLT2i administration on arterial stiffness has been little studied in T2DM patients particularly using the gold standard technique PWV and especially in conditions of 'real clinical practice' [9, 10]. Moreover, the clinical experiences on the influence of SGT2i on arterial stiffness brought conflicting results [11]. The aim of this case-control clinical experience was to explore the effects of adding an empaglifozin treatment for arterial stiffness in T2DM outpatients treated at a 3-month follow-up.

## 2 METHODS

The present study is a 12-week observational case-control clinical study performed from September 2018 to September 2019, at the Division of Endocrinology, Diabetes, and Metabolism of the Santa Croce e Carle Hospital in Cuneo (Italy). A total of 16 T2DM outpatients among those attending our diabetes outpatient clinic, were given empagliflozin (25 mg daily *per os*) according to current diabetes guidelines [12]; a control group formed by 16 consecutive T2DM patients was provided, in which empaglifozin was not added to the routine therapy over the same time period. Inclusion criteria were as follows: type 2 diabetes mellitus diagnosis, HbA1c > 6.5% (or > 47.6 mmol/mol), normal or mild decreased estimated glomerular filtration rate (eGFR) defined as eGFR > 60 ml/min/1.73m2, treatment-naive status with SGLT2i, absence of signs and symptoms of heart failure (HF), and a normal left ventricular systolic function. Exclusion criteria were as follows: type 1 diabetes mellitus diagnosis (or other types of diabetes mellitus), HbA1c < 6.5% (or < 47.6 mmol/mol), moderately or severely decreased eGFR defined as eGFR < 60 ml/min/1.73 m2 (or kidney failure), current treatment with SGLT2i, refusal to provide informed consent, challenges in assessing aortic stiffness, a lack of adherence to therapy, and frequent urinary tract infections. T2DM outpatients with HF or a left ventricular systolic dysfunction were excluded according to the strong suggestion to treat with SGLT2i in diabetes guidelines [12] and the influence of HF in the determination of PWV [13].

### 2.1 Endpoints

The objective of this case-control study was to analyze the impact of empagliflozin treatment, administered to outpatients with Type 2 Diabetes Mellitus (T2DM) during follow-up visits, on arterial stiffness and renal function after 12 weeks of treatment, in comparison to a control group of T2DM patients.

### 2.2 Study Protocol

A standardized evaluation form was utilized, encompassing the following components: demographic parameters (date of birth, age, and sex) and a detailed remote pathological history, which included a thorough assessment of traditional cardiovascular risk factors, such as smoking, diabetes mellitus, hypertension, and dyslipidemia. This evaluation was conducted for all enrolled patients. For inclusion in the study, an echocardiographic examination was provided as well as the arterial stiffness measurements as detailed hereafter. The evaluation of arterial stiffness has been controlled at a 3-month follow-up. Furthermore, venous blood and urine samples were collected at both the beginning and the end of the study for the following assays: Blood chemistry tests, including creatinine and Glycated hemoglobin (HbA1c), and the concentrations of urine albumin and creatinine, were determined using nephelometry and chromatography, respectively, in the clinical laboratory department of Santa Croce Hospital. The Albumin-to-creatinine ratio (ACR) (mg/g) was determined by dividing the concentration of albumin in the spot urine by the concentration of creatinine in the same urine sample. The estimated glomerular filtration rate (eGFR) was assessed with the MDRD formula expressed for use with standardized creatinine assay (5) as follows: GFR = 175 * standardized Scr -1.154 * age-0.203 *1.212 (if black) * 0.742 (if female). In this equation, GFR is expressed as ml/min per 1.73 m2, serum creatinine (Scr) is quantified in mg/dl, and age is calculated in years (14).

### 2.3 PWA and PWV Measurement

A non-invasive diagnostic tool was employed for the clinical evaluation of central arterial pressure to assess arterial stiffness parameters (SphygmoCor XCEL, AtCor Medical, Itasca, IL, USA). The SphygmoCor XCEL system measures the central aortic pressure waveform by detecting the pulsatile waves from the brachial artery cuffs. Analyzing this waveform yields critical parameters, such as central systolic pressure, central pulse pressure, arterial stiffness indices, augmentation pressure, and augmentation index. Changes in central systolic blood pressure and augmentation index have been identified as indicators of cardiovascular risk.

The SphygmoCor XCEL system also determines the pulse wave velocity (PWV) of the arterial pulse as it propagates from the descending aorta to the femoral artery. PWV is measured non-invasively by simultaneously capturing impulses from the carotid and femoral arteries. The carotid pulse is measured using a tonometer, while the femoral pulse is recorded *via* a cuff placed around the thigh. In subjects of a certain age, normal PWV values are generally considered to be below 9–10 m/s. An increase in aortic stiffness or evidence of target organ damage may be indicated by a rise in the velocity of the carotid and femoral impulses.

Measurements should be conducted with the patient in a supine position in a quiet environment. Smoking within one hour prior to the exam, excessive intake of vasoactive substances (*e.g.,* coffee), and interruption of pharmacological therapy should be avoided. The Augmentation Index (AIx75) is calculated at the carotid artery by recording ten high-quality pulse wave samples. Proprietary software from the manufacturer automatically computes the Aix, which reflects the pressure increase caused by the return of reflected waves in the aorta [14, 15].

In line with the most common guidelines [16], all echocardiograms were performed using a GE Vivid 7 Pro. For volumetric measurements, two-dimensional apical views in both 2- and 4-chamber orientations were utilized. Left ventricular ejection fraction (LVEF) was determined using a modified Simpson’s method based on the biplane apical views. Data on left ventricular end-diastolic and end-systolic volumes were collected. All echocardiograms were performed by expert operators who were blinded to the BNP assay results, with intra-observer variability for LVEF assessment being under 5%. The echocardiographic measurements included left ventricular end-diastolic diameter, as well as the diastolic thickness of the interventricular septum and the posterior wall of the left ventricle. LVEF values below 50% were deemed indicative of left ventricular systolic dysfunction.

This analysis was conducted in accordance with the ethical principles of the Declaration of Helsinki and adhered to Good Clinical Practice guidelines.

In particular, this research was conducted on humans by the Helsinki Declaration of 1975, as revised in 2013 (http://ethics.iit.edu/ecodes/node/3931).

The study protocol was approved by the institutional review board and independent ethics committee of our institution (Ethic Committee of Mondovì Hospital, Italy, n.10-18, September, the 12^th^, 2018). All patients provided written informed consent for treatment and arterial stiffness measurements.

### 2.4 Statistical Analysis

Data are presented as mean values ± standard error of the mean (SEM), unless otherwise specified, or as medians with interquartile ranges, or as percentages. The Shapiro-Wilk test was employed to evaluate the normality of data distribution. Depending on the characteristics of the data, either the Wilcoxon signed-rank test or the paired Student’s t-test was utilized to compare continuous variables. To assess differences in arterial stiffness between the two groups, an analysis of Covariance (ANCOVA) was performed, controlling for age and HbA1c levels as covariates. Differences in categorical variables were analyzed using either the χ^2^ test or Fisher’s exact test, as appropriate. A significance level of *P* < 0.05 was established. All calculations were conducted using STATISTICA for Windows, version 5.1 (Statsoft Inc., Tulsa, OK, USA).

## 3 RESULTS

Thirty-two outpatients with T2DM completed this clinical experience. Table **1** presents the characteristics of the patients. The duration of diabetes mellitus and the distribution of sex did not differ between groups. Patients treated with empagliflozin were younger in comparison to controls (*p* = 0.002). There was only one smoker in the empagliflozin-treated group and two in the control group. Renal function, the duration of diabetes illness, as well as cardiac parameters, were similar in the two groups. There were no significant differences in diabetes treatment observed between the two groups. Both groups were primarily treated with metformin, and there were no notable differences in the use of GLP-1 receptor agonists (GLP-1 RAs), DPP-4 inhibitors, or insulin. Additionally, 14 out of 16 participants in the empagliflozin group and 15 out of 16 in the control group were administered antihypertensive medications, with a predominant use of ACE inhibitors, angiotensin receptor blockers, or calcium channel blockers (the percentage of patients using ACE inhibitors, angiotensin receptor blockers, calcium antagonists, and beta-blockers was similar in the two groups; however, the data are not reported in the table). Statins or ezetimibe were provided to 14/16 and 15/16 patients, respectively. The results of the follow-up have been described in Table **2**. At the 3-month follow-up, empagliflozin treatment proved to significantly reduce HbA1c (*p* = 0.007) (Table **2**), while an increase was observed in the control group (*p* = 0.004). The body weight, as expected, and BMI were reduced in the empaglifozin group (*p* = 0.001 for both), while they were increased in controls (*p* = 0.01 for body weight). In the two groups, estimated GFR remained unchanged, while the urine albumin-to-creatinine ratio showed a significant improvement following treatment with empagliflozin (*p* = 0.049).

In comparison to controls, empagliflozin significantly reduced PWV (*p* = 0.005 for the difference; ΔPWVV -0.68 ± 1.1 *vs* 0.89 ± 1,6 *p* < 0.004, *p* = 0.0065 with age and HbA1c as covariates) at 3-month follow-up (Table **2**).

## 4 DISCUSSION

In this real-world clinical study, we explored the potential effects of empagliflozin treatment on arterial function in patients with Type 2 Diabetes Mellitus (T2DM). This experience highlights two main considerations:

a) Pulse wave velocity (PWV), the primary parameter defining arterial stiffness, significantly improved following empagliflozin treatment after a mid-term follow-up. Several previous studies have examined the effects of SGLT2 inhibitors on arterial stiffness. In young patients with Type 1 Diabetes (T1DM), treatment with 25 mg of empagliflozin daily resulted in a significant reduction in systolic blood pressure and various arterial stiffness parameters after 8 weeks. This included improvements in augmentation indices at the radial, carotid, and aortic levels, as well as the carotid-radial pulse wave velocity (PWV) [17]. Levey *et al.* also observed similar effects in T2DM patients who were treated with 25 mg of empagliflozin daily for six weeks [14]. It is worth noting that other anti-diabetic drugs, such as metformin, have demonstrated a clear protective effect on endothelial function in several studies [17, 18]. In our study, however, blood pressure was not affected by empagliflozin treatment. This result is likely due to the small sample size and the minimal changes in pressure that may have been undetectable. Furthermore, a pilot study discovered that flow-mediated dilation (FMD) improved and aortic pulse wave velocity (PWV) decreased after just two days of treatment with 10 mg of dapagliflozin daily in patients with type 2 diabetes mellitus (T2DM) [10].

Moreover, the lack of difference between the two groups in terms of AIx75 may be related to the limited sample size. Expanding the sample size could provide further insight into the potential effects of empagliflozin treatment on the augmentation index.

To our knowledge, this is the first study to explore the effects of empagliflozin on arterial function in T2DM outpatients using the gold-standard method of Pulse Wave Velocity (PWV) in real clinical practice. Actually, a study in mice by Aroor *et al.* provided compelling evidence on the beneficial effects of the SGLT2 inhibitor empagliflozin, particularly in reducing aortic stiffness and renal resistivity index, which have significant implications for vascular health and kidney function [19-24].

The improvement in arterial stiffness observed with empagliflozin could be attributed to a reduction in acute oxidative stress, potentially explaining the drug's sustained effect, as seen in our study. Furthermore, its anti-inflammatory properties may increase the bioavailability of nitric oxide (NO) within the arterial wall. Treatment with empagliflozin also led to significant improvements in microvascular reactivity, aligning with findings from similar studies involving patients with type 2 diabetes mellitus (T2DM). While many studies have explored the positive effects of empagliflozin and dapagliflozin on arterial stiffness [9-11], these have primarily focused on T2DM, with only one study involving T1DM patients [19]. Our findings align with previous studies, reinforcing these observations. Although metformin has been associated with improvements in endothelial function [16], its impact on arterial stiffness appears to be limited [20]. It can be hypothesized that specific receptor/signaling pathways activated by SGLT2 inhibitors, such as gliflozin, contribute to these improvements [21]. Evidence indicates that SGLT2 receptors are located on vascular smooth muscle cells (VSMCs) [21], although their clinical significance remains a topic of debate.

Overall, it appears plausible that empagliflozin uniquely activates the reduction of arterial stiffness, potentially by acting on SGLT2 receptors in VSMCs. These effects are likely to enhance cardiovascular protection, as evidenced by clinical studies [22].

Furthermore, the role of glucolipotoxicity in exacerbating insulin resistance has been well-documented, with studies indicating that elevated free fatty acids and glucose levels contribute to vascular stiffness. Pathological changes in the vascular bed, driven by glucolipotoxicity, could serve as critical endpoints in evaluating the efficacy of empagliflozin. The relationship between insulin resistance and vascular stiffness should be given more attention, particularly regarding its implications for treatment strategies with empagliflozin in diabetic patients. This could lead to a better understanding of cardiovascular risk management in this population [25].

b) The improvement in the urine albumin-to-creatinine ratio (UACR) suggests a betterment in endothelial function, potentially associated with the observed decrease in arterial stiffness. The significant decrease in UACR among patients treated with empagliflozin suggests notable improvements in endothelial health. Elevated UACR is frequently an indicator of renal microvascular damage and endothelial dysfunction, both of which are crucial factors in the onset of cardiovascular disease and diabetic kidney disease [23].

As a sodium-glucose cotransporter 2 (SGLT2) inhibitor, empagliflozin seems to offer endothelial protection through mechanisms including enhanced glycemic control, decreased inflammation, and modulation of hemodynamics. In our exploration of empagliflozin's mechanisms, we underscore its promising role in mitigating inflammation and endothelial dysfunction, consistent with the findings of Papaetis, who highlighted the drug's multifaceted effects on diabetic kidney disease [26, 27]. By promoting natriuresis and diuresis, empagliflozin may alleviate extracellular fluid overload, thereby reducing arterial stiffness—a critical factor in cardiovascular risk. The reduction in UACR not only indicates improved renal function but also supports the notion of reduced arterial stiffness, which is closely tied to endothelial function. Healthier endothelium results in improved vascular responsiveness, lower systolic blood pressure, and overall enhanced cardiovascular outcomes.

## CONCLUSION

Empagliflozin, when added to anti-diabetic treatment for type 2 diabetes mellitus, significantly enhanced arterial stiffness during a mid-term follow-up. Empagliflozin significantly improved arterial stiffness, with changes noted within clinically relevant ranges. In conclusion, the significant improvement in UACR seen with empagliflozin therapy underscores its role in promoting endothelial health and may plausibly contribute to the reduction of arterial stiffness, highlighting the therapeutic potential of SGLT2 inhibitors in managing both renal and cardiovascular risks in patients, particularly those with diabetes. Such findings could bolster the cardioprotective and nephroprotective effects observed in this and other SGLT2 inhibitors in extensive clinical studies [26-29].

## LIMITATIONS OF THE STUDY

The main limitation seemed to be the reduced population studied. The small sample size presents some limitations for drawing more definitive conclusions; however, we believe it is adequate for the primary endpoints. The age difference between the two groups, participants in the empagliflozin group and those in the control group, was significantly different. Recognizing that age can influence present results, we employed ANCOVA to control this variable. After adjusting for age, our analysis revealed that empagliflozin treatment maintained a statistically significant effect on arterial stiffness, indicating that the benefits of empagliflozin treatment are robust across different age groups. These findings suggest that regardless of age differences, empagliflozin treatment demonstrates a clear benefit, which is an important consideration for clinical applications. Future research may explore this effect across a broader age spectrum to further validate these results. This statistical approach ensures clarity and rigor in explaining the significance of the results while addressing age as a potential confounder in the analysis. Moreover, at the time of the study, limitations imposed by Italian pharmacological regulatory agencies regarding glomerular filtration rate (GFR) influenced the age of the patients included in the treatment group.

## Figures and Tables

**Table 1:** Patient characteristics in study groups at inclusion in the study (baseline).

-	**Empagliflozin (16 pts) **	**Controls (16pts)**	***p***
**Age ( years)**	63.9 ± 2.0	73.8 ± 1.9	<0.002
**Sex (male/female)**	11/6	10/6	ns
**BMI (kg/m2)**	30.5 ± 1.4	27.3 ± 2.9	ns
**Diabetes Duration ( years)**	9.7 ± 1.7	9.6 ± 1.4	ns
**HbA1c (%) **	7.9 ± 0.2	7.2 ± 0.3	ns
**eGFR (ml/min/.73m^2^)**	87.4 ± 25.5	105.4 ± 42.3	ns
** SBP (mmHg) **	143.1 ± 20.2	147.3 ± 23.1	ns
** DBP (mmHg) **	81.7 ± 9.4	84.7 ± 11.2	ns
** Total cholesterol (mg/dl) **	167.3 ± 37.2	170.4 ± 30.5	ns
** HDL Cholesterol (mg/dl) **	40.6 ± 7.3	42.2 ± 7.7	ns
** Triglycerides (mg/dl) **	143 (100-221)	110 (70-112)	ns
** LDL Cholesterol (mg/dl) **	96.0 ± 20.6	98.5 ± 28.7	ns
**LVEF (%)**	57.6 ± 7.9	59.3 ± 11.9	ns
**LVESD (mm)**	29.7 ± 6.6	33.7 ± 5.7	ns
**LVEDD (mm) **	47.1 ± 4.7	51 ± 5.9	ns
**Sinus rhythm **	10/16	13/16	ns

**Note:** Values are expressed as mean  ±  standard error of the means, except for the triglyceride values expressed as mean and interquartile range. **Abbreviations:** BMI = body mass index; HbA1c = glycated haemoglobin; eGFR = glomerular filtration rate; LVEF = left ventricular ejection fraction; LVESD = left ventricular end-systolic diameter; LVEDD = left ventricular end-diastolic diameter.

**Table 2:** Differences in main variables after 3-month treatment with empaglifozin and in the control group.

-	**Empaglifozin Baseline**	**Empaglifozin Follow-Up**	**Controls Baseline **	**Controls ** **Follow-Up**
**eGFR (ml/min/.73m^2^)**	87.4 ± 25.5	90.6 ± 19.3	105.4 ± 42.3	99.8 ± 21.7
**ACR**	17.8 ± 46.8	12.2 ± 35.7	2.1 ± 1.9	1.4 ± 0.7
**PWV (m/s) **	13.2 ± 2.0	12.3 ± 1.8**	12.8 ± 2.3	13.2 ± 2.4
**HbA1c (%)**	7.9 ± 0.78	7.04 ± 1.1**	6.54 ± 0.4	7.14 ± 0.5**
**Body weight (kg)**	86.7 ± 16.1	81.7 ± 16.5**	86.2 ± 19.3	87.7 ± 19.2*
**BMI (kg/m2)**	30.5 ± 5.4	28.7 ± 5.6**	24.4 ± 0.8	24.8 ± 1.1
**Creatinine (mg/dl) **	0.91 ± 0.24	0.86 ± 0.23	0.98 ± 0.17	0.91 ± 0.15
**U Albumin/creatinine (mg/mmol)**	17.8 ± 46.8	12.2 ± 35.7*	13.2 ± 11.8	46.5 ± 79.4
**AIx 75 **	34.3 ± 9.9	35.8 ± 9.4	34.3 ± 9.9	32.9 ± 9.5

**Note:** Values are expressed as mean  ±  standard error of the means. *p < 0.05; **p < 0.01. **Abbreviations:** eGFR = glomerular filtration rate; ACR = Albumin/Creatinien Ratio; PWV = pulse wave velocity; HbA1c = glycated haemoglobin; BMI = body mass index; AIx75 = augmentation index.

## Data Availability

The data and supportive information are available within the article.

## LIST OF ABBREVIATIONS

T2DM: Type 2 Diabetes Mellitus
CVD: Cardiovascular Disease
SGLT2 inhibitors: Sodium-Glucose Cotransporter 2 Inhibitors
PWV: Pulse Wave Velocity
eGFR: Estimated Glomerular Filtration Rate
HF: Heart Failure
ACR: Albumin-to-Creatinine Ratio
eGFR: Estimated Glomerular Filtration Rate
Scr: Serum Creatinine
AIx75: Augmentation Index
LVEF: Left Ventricular Ejection Fraction
SEM: Standard Error Mean
ANCOVA: Analysis Covariance
(FMD): Flow-mediated Dilation
NO: Nitric Oxide
VSMCs: Vascular Smooth Muscle Cells
UACR: Urine Albumin-to-creatinine Ratio

## STANDARDS OF REPORTING

Strobe guidelines were followed.
